# An automated tool for localization of heart sound components S1, S2, S3 and S4 in pulmonary sounds using Hilbert transform and Heron’s formula

**DOI:** 10.1186/2193-1801-2-512

**Published:** 2013-10-05

**Authors:** Ashok Mondal, Parthasarathi Bhattacharya, Goutam Saha

**Affiliations:** Department of Electronics and Electrical Communication Engineering, Indian Institute of Technology, Kharagpur, Kharagpur, 721 302 India; Institute of Pulmocare and Research, Kolkata, Kolkata, 700 064 India

**Keywords:** Filtering; Heart sound localization; Hilbert transform; Heron’s formula; Lung sound

## Abstract

**Abstract:**

The primary problem with lung sound (LS) analysis is the interference of heart sound (HS) which tends to mask important LS features. The effect of heart sound is more at medium and high flow rate than that of low flow rate. Moreover, pathological HS obscures LS in a higher degree than normal HS. To get over this problem, several HS reduction techniques have been developed. An important preprocessing step in HS reduction is localization of HS components. In this paper, a new HS localization algorithm is proposed which is based on Hilbert transform (HT) and Heron’s formula. In the proposed method, the HS included segment is differentiated from the HS excluded segment by comparing their area with an adaptive threshold. The area of a HS component is calculated from the Hilbert envelope using Heron’s triangular formula. The method is tested on real recorded and simulated HS corrupted LS signals. All the experiments are conducted under low, medium and high breathing flow rates. The proposed method shows a better performance than the comparative Singular Spectrum Analysis (SSA) based method in terms of accuracy (ACC), detection error rate (DER), false negative rate (FNR), and execution time (ET).

## Introduction

The conventional stethoscope based auscultation technique is a cost-effective and non-invasive diagnostic procedure. This technique is very popular among the physicians and commonly used by them since 1816 (Laennec 1962). However, the performance of this diagnostic technique degrades due to the presence of noise in lung sound signals. Modern electronic stethoscope can reduce the ambient noises from lung sounds, but they are inefficient to avoid heart sound noise.

During the recording of lung sound, heart sound interferes and changes the temporal and spectral contents of the respiratory sound. This may lead to misinterpretation of the underlying lung diseases. Heart sounds comprise of two primary sounds S1 and S2. In addition, it may have components like S3, S4 and murmurs associated with pathological conditions. The first heart sound, S1 and second heart sound, S2 are produced by the openings of the atrioventricular valves and closures of semilunar valves, respectively and vice versa (Pourazad 2004). The third (S3) HS occurs at the end of S2 due to the vibration of blood inside the ventricles and the fourth (S4) HS is appeared just before the S1 due to the contraction of atria (Webster 1998; Balasubramaniam and Nedumaran 2010). These components carry important information regarding the cardiac system and are segmented to diagnose the valvular heart diseases (Schmidt et al. 2010; Tang et al. 2012; Sanei et al. 2011; Patidar and Pachori 2013). However, lung sounds are produced by stochastic and disruptive flow of air within lung airways (Blake 1986). Most of the heart sound information lies in the frequency range of 20-150 Hz (Arnott et al. 1984; Lu et al. 1988; Cromwell et al. 2002) but murmur sounds have a higher frequency range of 600 Hz (Patidar and Pachori 2013). On the other hand, lung sound information spread out over a wide range of frequency approximately 20-1600 Hz (Gavriely et al. 1981). However, a major part of the lung sound information is confined to a frequency less than 200 Hz (Sovijarvi et al. 2000).

With the advances in modern technologies, computer science, and statistical signal processing, a lot of research work has been conducted to overcome the problem of HS removal to highlight the LS (Iyer et al. 1986; Lu et al. 1988; Kompis and Russi 1992; Hadjileontiadis and Panas 1997; Gnitecki et al. 2003; Gnitecki and Moussavi 2003; Ahlstrom et al. 2005; Yadollahi and Moussavi 2006; Pourazad et al. 2006; Flores-Tapia et al. 2007; Ghaderi et al. 2011). This is an important preprocessing step in lung sound analysis. A common approach of heart sound reduction is high pass filtering of lung sounds. However, this approach attenuates the lung sound components that resemble to heart sound in spectral domain (Donoho 1995). Except the wavelet de-noising technique, the performance of all the other heart sound cancellation methods depends on a properly defined heart sound location. Many research groups have developed methods to detect the heart sounds locations in LS signals. These methods are based on adaptive filtering (Iyer et al. 1986; Lu et al. 1988; Kompis and Russi 1992; Hadjileontiadis and Panas 1997; Gnitecki et al. 2003), time-frequency filtering (Pourazad et al. 2006), multiscale product based (Flores-Tapia et al. 2007), and statistical signal analysis (Gnitecki and Moussavi 2003; Ahlstrom et al. 2005; Yadollahi and Moussavi 2006; Ghaderi et al. 2011) approaches. Adaptive filtering techniques need a reference heart sound signal which is produced either by the noisy lung sound signal itself or by an external source, e.g., electrocardiogram (ECG) signal. A combined theory of spectrogram and wavelet transform analysis is proposed to detect the HS components in (Pourazad et al. 2006). A multiscale product based method is implemented in (Flores-Tapia et al. 2007) to localize the HS segments. Several statistical methods are used to find out the heart sound locations, such as variance fractal dimension trajectory (Gnitecki and Moussavi 2003), recurrence time statistics (Ahlstrom et al. 2005), entropy (Yadollahi and Moussavi 2006), and singular spectrum analysis (SSA) (Ghaderi et al. 2011). Entropy (ENT) and SSA based algorithms give comparatively better results than other techniques. However, SSA method gives better results than that of ENT method in terms of false negative rate, error in localization and correlation coefficient. Moreover, SSA method is much faster than ENT method. Gadheri et al. has shown the superiority of SSA method over ENT technique in (Ghaderi et al. 2011).

All these techniques highlight their performances for normal lung sound signals at low and medium flow rate but not at high flow rate.

The objective of this work is to develop an effective and efficient algorithm to localize primary heart sounds (S1 and S2) and pathological heart sounds (S3 and S4) components that is applicable to both normal as well as abnormal cases of lung sounds for three different breathing flow rates: low, medium and high. In this paper, a novel heart sounds (S1, S2, S3 and S4) localization algorithm is proposed by using Hilbert transform (Johansson 1999; Mertins 1999) and Heron’s formula (Stanojevié 1997) and the proposed method is referred to as Hilbert Heron Algorithm (HHA). The HS and non HS segments are discriminated by investigating the morphological characteristics of the cardiac sounds. It has been taken under consideration that each HS component extends for a certain duration (Khandpur 2003; Schlant and Alexander 1994) and defined by two global minima and one maximum points. The minima points are correspond to the starting and ending of the HS component and the maximum point is correspond to the highest energy peak of the HS component. By connecting the global extrema points some scalene triangles can be drawn and their areas will be used to identify the HS and non HS segments. The area of the triangle can be computed from the envelope signal using Heron’s formula. The envelope signal is estimated from the filtered mixed LS signal using HT. The decision regarding the heart sound included segment or heart sound excluded segment is taken by comparing the area with an adaptive threshold value. The threshold value is calculated from the variance of the area vector as discussed in Section "Methodology". The performance of the proposed method is compared with the SSA method by evaluating the results for both cases of simulated mixed lung sound signals (normal and pathological) and real recorded lung sound signals. The method gives better performance than the SSA method in terms of false negative rate, accuracy, detection error rate, and execution time.

The remaining part of this paper is organized as follows. Section "Theoretical background on Hilbert transform and Heron’s formula" provides theoretical background on the Hilbert transform and Heron’s formula and Section "Methodology" describes in detail the methodology. The experimental database and certain implementation issues are described in Section "Experimental data sets and implementation issues" and Section "Results and discussion" presents the experimental results and discusses the efficiency of the method. The conclusion is given in section "Conclusion".

## Theoretical background on Hilbert transform and Heron’s formula

### The Hilbert transform

Hilbert transform was developed by German scientist David Hilbert (Johansson 1999; Mertins 1999) in the beginning of the 20th century for interpreting the Euler formula1

where *j* is an imaginary unit, i.e., . The Hilbert transform of a real valued continuous time domain signal, *y*(*t*) is defined by2

where *s* is real and *H*{·} is the Hilbert operator. Here, the integration has to be carried out according to the Cauchy principle value, that is,3

However, real world signals are discontinuous in nature and can be expressed as a discrete-time signal. In the discrete domain, the envelope of the real discrete signal *y*[*n*] is estimated by the discrete Hilbert transform denoted by *H*_*d*_{·}. The discrete Hilbert transform *H*_*d*_{·} of a sequence *y*[*n*] having a finite period *R* can be computed using its Discrete Fourier transform (DFT). The DFT of *y*[*n*] is denoted by *Y*[*m*] is calculated by4

where , *m* is the discrete frequency and *n* is the discrete time. The DFT *Y*[*m*] of the discrete time domain signal *y*[*n*] can be expressed as a combination of a real and an imaginary component, i.e.,

The discrete Hilbert transform  of *y*[*n*] is calculated as56

The equation (5) is applicable when *R* is even and equation (6) for odd *R*.

### Heron’s formula

Heron was a Greek mathematician and engineer in 10-70 AD (Stanojevié 1997). He contributed much in the field of optics, mathematics and enginering. But Heron is popular for deriving the formula for computing the area of the triangle. The formula consists of two steps:

Step 1: Compute the semiperimeter h of the triangle using the lengths of its sides, u, v, and w as7

Step 2: Calculate the area A of the triangle using semiperimeter and the lengths of its sides by the equation (8).8

where

The detail of the formula is given in proposition 1.8 of his book, Metrica. The proof of Heron’s formula has been done by Roger Boscovich and stated in (Stanojevié 1997).

## Methodology

**Figure 1:**
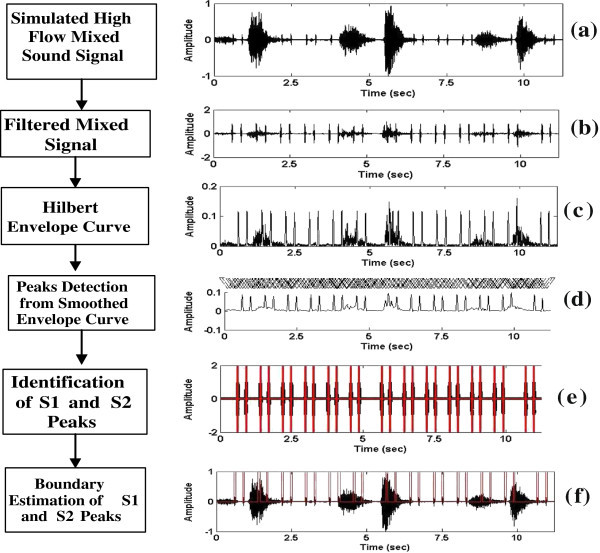
**Flow chart of the proposed method.** Sub-figures **(a)**-**(f)** depict the outcomes of the different steps of the proposed method. The sound signal displayed in **(a)** corresponds to high flow mixed signal. The white arrowheads in **(d)** indicate the the events that correspond to HS and LS components.

The proposed method distinguishes HS element from non HS element based on the principle that heart sound component has higher area than that of the non heart sound component. A flow chart of the proposed method is given in Figure 1. The entire process is comprised of the following steps:

### Amplitude normalization

The amplitude of the mixed signal varies considerably due to various factors such as recording instrument gain, different recording locations over the anterior and posterior chest, physiology, age and sex of the subject. The amplitude variation creates a difficulty during analysis of different signals of non-uniform amplitude. This problem is minimized by setting the maximum value of variance of the signal to +1 and minimum to -1. Let *y*(*n*) be the signal value at *nth* sample, and *M* be the absolute maxima in the sample space. The normalized signal *y*_*norm*_(*n*) is given by910

Here *n* = 1,2,3,…,*K* and *K* is the total number of samples in the signal.

### Filtering

**Figure 2:**
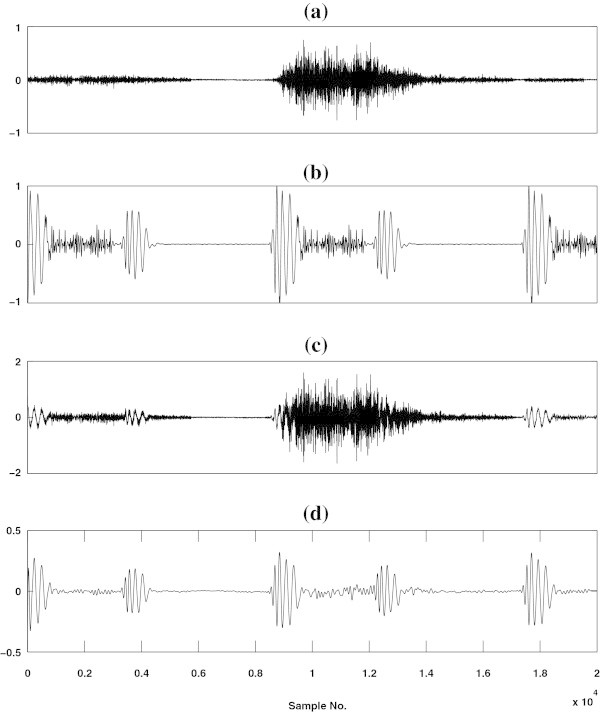
**(a) A typical example of a normal LS signal.** **(b)** A time domain representation of an abnormal HS signal (pan systolic murmur). **(c)** Wave form of the mixed signal and **(d)** wave form of the filtered signal.

The intensity and frequency components of the pulmonary sound change according to the variation in flow rate. The amplitude of the lung sound increases with an increase in respiratory flow (Yadollahi and Moussavi 2006). Hence, HS may become invisible for medium and high flow rates. It is very difficult to localize the HS segments for high flow than medium and low flow cases. In this study, the flow rate effect is minimized by filtering the mixed lung sound signals using a 10*th* order Butterworth finite impulse response (FIR) filter with a cutoff frequency of 150 Hz. The filtering operation enhances the HS components by attenuating the higher frequency LS and murmur components as shown in Figure 2. The filtered sound is used as input to the next step to detect the HS segments.

### Hilbert Envelope Extraction

The envelope curve gives a simple and demonstrable representation of a narrow band signal to investigate its intrinsic characteristics (Choi and Jiang 2008). The envelope of a signal can be computed by different techniques like Hilbert transform and Shannon energy based approach. In this study, we have chosen HT to compute envelope of HS dominated filtered mixed sound signal because of two reasons. Firstly, Shannon energy based envelope detection algorithm performs well for medium intensity signal and it gives poor result for high and low intensity signal (Choi and Jiang 2008; Ari and Saha 2007). Secondly, the computational complexity of Shannon envelope is much greater than that of the Hilbert envelope because Shannon method takes into considerations the windowing and overlapping processes (Choi and Jiang 2008; Ari and Saha 2007). We have experimentally observed that HT takes only 0.02 sec for calculating the Hilbert envelope and Shannon energy based method takes 3.97 sec for computing the Shannon envelope for the same signal of length 40313 samples. As lung sound shows a wide band spectrum and heart sound gives a narrow band spectrum, the Hilbert transform facilitates to detect the heart sound elements in lung sound signals in spite of their spectral overlap. Hilbert envelope can trace the variation of heart sound components in lung sound as a periodic train. The Hilbert envelope is computed based on Hilbert Transform as described in Section "Theoretical background on Hilbert transform and Heron’s formula". Let *x*_*nf*_(*n*) be the normalized, filtered mixed signal. The complex analytic signal *A*[*x*_*nf*_(*n*)] of the given signal *x*_*nf*_(*n*) is expressed as11

The envelope curve *E*_*H*_(*n*) of the given signal *x*_*nf*_(*n*) can be computed from the magnitude of the analytic signal *A*[*x*_*nf*_(*n*)], and is expressed as12

The phase *ϕ*(*n*) information of the analytic signal *A*[*x*_*nf*_(*n*)] is determined by the following equation13

### Peak Detection in the Envelope

The Hilbert envelope curve *E*_*H*_(*n*) is estimated from the filtered mixed signal using equation (12) and is shown in Figure 1(c). The envelope signal consists of many peaks which are originated from the HS components and from the low frequency LS components of the filtered mixed signal as shown in Figure 1(b). Each peak of the envelope curve *E*_*H*_(*n*) has a rising and a falling edges, respectively. The rising edge gives the positive gradient values and falling edge gives negative gradient values at each point over the envelope. These peaks are detected through the following steps:

Step 1: Smoothening of the envelope: The Hilbert envelope *E*_*H*_(*n*) of the signal is not smooth because of the presence of lung sound components. Hence, it is required to smoothen for more accurate peak detection which is associated with HS. To accomplish this, a filtering operation is done using a 5*th* order Butterworth FIR filter with a cutoff frequency varying in a range of 7-25 Hz. We discuss the effect of variation in cut-off frequency in Section "Results and discussion".

Step 2: Identification of local maxima and minima: The extreme points of the envelope signal can be calculated by considering the sign changes across the first derivative of the envelope. A sample value *E*_*H*_(*i*) of the smoothed envelope curve will be a minimum valued point for  and will be a maximum valued point for .

Step 3: Estimation of peaks: A peak consists of three consecutive extrema points which include two minima and one maximum. Each peak has a finite extension from one minimum point to another as shown in Figure 1(d). The duration of the individual peak varies according to its source characteristics. The peak locations are identified by calculating their extreme points and marked with a white arrow head in Figure 1(d).

### Picking up the S1, S2, S3 and S4 peaks

The peaks detected using the above described peak detection framework do not always correspond to heart sound components. Some peaks occur due to the presence of artifacts and unfiltered lung sound components. The non-heart sound peaks are rejected and the heart sound peaks are selected using a geometrical formula derived by Greek mathematician Heron.

Selection criteria of S1, S2, S3 and S4 peaks: The area of individual peak is calculated using Heron’s triangular formula. The triangles are formed by connecting the extreme points of the peaks. Let us consider the minima and maximum points for *ith* peak are , , and , respectively. The length of each side of the triangle associated with *ith* peak are calculated as follows:14

where *c*^*i*^ is the base, *a*^*i*^ is the left lateral side and *b*^*i*^ is the right lateral side of the triangle. The three angles of the triangle are defined by the following equation15

where *α*^*i*^, *β*^*i*^ and *γ*^*i*^ are the angles between the three sides. The lengths of the three sides of the triangle are unequal in magnitude and the angles in between them are also unequal in degree. Hence this triangle satisfies the criterion of scalene triangle. The area △^*i*^ of the *ith* triangle is calculated as161718

where *i* indicates the number of triangle and lies in the range defined by 1 ≦ *i* ≦ Γ-2 × *Q*, *P* and *Q* are the total number of minima and maximum points in the envelope, respectively. The area of heart sound components is higher than that of the artifacts or low frequency lung sound components because heart sound components have a high peak amplitudes as shown in Figure 1(b). The heart sound components S1, S2, S3 and S4 are identified by comparing the area of the peak with an adaptive threshold value that is calculated from the variance *σ* of the area vector **A** = [*A*_1_,*A*_2_,*A*_3_,…,*A*_*Q*_]^*T*^, where *A*_*r*_(*r* = 1,2,…,*Q*) indicates the area of individual peak in corrupted LS. The heart sound peaks *P*_*HS*_ are selected using the Algorithm 1.

**Algorithm 1** Calculate *P*_*HS*_

### Boundary estimation of S1, S2, S3 and S4 peaks

The primary HS components (S1 and S2) extend on both sides of its peak position as shown in Figure 1(e) for a finite length due to the time gap between the closures and openings of the heart valves (Pourazad 2004) but the third (S3) and fourth (S4) HS components extend due to the relaxation of the ventricle and atrium heart chambers (Webster 1998; Balasubramaniam and Nedumaran 2010). To estimate the HS boundary, peak location identification is needed. The peak locations are detected using Algorithm 1, and after that their boundary *B*_*HP*_ are calculated by using Algorithm 2.

**Algorithm 2** Calculate *B*_*HP*_

## Experimental data sets and implementation issues

### Subjects and data acquisition

The lung sound signals are recorded from the normal as well as abnormal male and female subjects using a single channel stethoscope based data acquisition system as described in (Mondal et al. 2011). The data acquisition system has been constructed by making a circuit using active devices (Transistors. Operational Amplifiers) and passive elements (Resistors, Capacitors and Inductors) fitted to a stethoscope to capture the LS using the diaphragm mode. The LS data are recorded from different auscultation locations over the body surface (e.g., left mid clavicular area, 2nd intercostal and third intercostal spaces) of the patients in the sitting position and under relaxing mood conditions. The recordings are not associated with any particular age group. The recorded data are arranged in 16 bit, PCM, Mono audio format and stored as *.wav files at sampling frequency of 8 kHz. The pathological LS are recorded from 8 female and 20 male subjects with different types of pulmonary dysfunctions: Chronic Obstructive Pulmonary Diseases (COPDs), Interstitial Lung Disease (ILD) and asthma. The pathological HS are recorded from 10 female and 22 male subjects with various valvular heart diseases. On the other hand, the normal LS are recorded from 5 male healthy subjects and normal HS from 3 female and 5 male subjects. The pulmonary sound records are collected from various resources: Institute of Pulmocare and Research, Kolkata, Audio & Biosignal Processing laboratory, IIT Kharagpur, India and also from R.A.L.E. dataset available at: http://www.rate.cal. The cardiac sound data are collected from the two institutes mentioned above and also from the Maulana Azad Medical Institute, Delhi, India. The abnormal lung sounds include wheezes, crackles and squawks sounds and abnormal heart sounds include late systolic murmur, pulmonary stenosis, early systolic murmur, ejection click, aortic insufficiency, pan systolic murmur, etc.

### Synthetic data

The synthesized mixed lung sound data at different flow rates are generated by a convoluting mixture producing technique as described in (Ghaderi et al. 2011). The convolutive mixtures are simulated by imposing the filtered heart sound components *FS*_*HS*_(*t*) onto the filtered lung sound components *FS*_*LS*_(*t*) as given next.192021

where *a*_*p*_ and *b*_*q*_ are the vectors of lung sound and heart sound filter coefficients, respectively. These are four dimensional vectors, i.e., **a**_*p*_ = [*a*_*p*0_,*a*_*p*1_,*a*_*p*2_,*a*_*p*3_]^*T*^ and **b**_*q*_ = [*b*_*q*0_,*b*_*q*1_,*b*_*q*2_,*b*_*q*3_]^*T*^. The heart sound filter coefficient vector **b**_*q*_ is normalized to one, i.e., ∥**b**_*q*_∥ = 1. These filter coefficients are generated randomly.

The high, medium and low flow rates mixed signal are synthesized by varying the amplitude ratios of the heart and lung sound signals. In this study, four types of mixtures at three different flow rates are examined. The range of the norm of the lung sound filter coefficients vector **a**_*p*_ corresponding to different flow rates for various types of mixtures are given in Table 1. The classifications of flow rates have been done empirically by a pulmonologist.

**Table 1:** **The values of norms for different flow rates**

TLS	THS	TM	Range of Norm	TF
			of ***a***_***p***_	
			> 3.10	High
Normal	Normal	Normal	0.81-3.10	Medium
			0.10-0.80	Low
			> 3.30	High
Normal	Abnormal	Abnormal	0.91-3.30	Medium
			0.10-0.90	Low
			>3.28	High
Abnormal	Normal	Abnormal	1.59-3.28	Medium
			0.10-1.58	Low
			> 3.35	High
Abnormal	Abnormal	Abnormal	1.97-3.35	Medium
			0.10-1.96	Low

TLS: Types of Lung Sounds; THS: Types of Heart Sounds; TM: Types of Mixtures; TF: Type of Flow.

### Implementation platform

The whole analysis is implemented on an ACER-PC with 3.29 GHz Intel core 2 quad CPU and 3.49 GB of RAM. The MATLAB (R2008a, The Mathworks, Inc., Natick, MA) tool is used for conducting the all experiments.

## Results and discussion

The efficiency of the proposed method is measured by evaluating the results in terms of false positive rate (FPR), false negative rate (FNR), accuracy (ACC), detection error rate (DER), and execution time (ET), and compared with the SSA method. These performance measuring units are calculated by the following equations:22232425

False negative (FN) occurs when HS segment is missed and false positive (FP) occurs due to misidentification of non HS as HS. On the other hand, true negative (TN) and true positive (TP) occur when HS segment and LS segment are correctly detected. All the experiments are conducted on the same database mentioned in Section "Experimental data sets and implementation issues" with the proposed and SSA method.

### Graphical interpretation of the results

**Figure 3:**
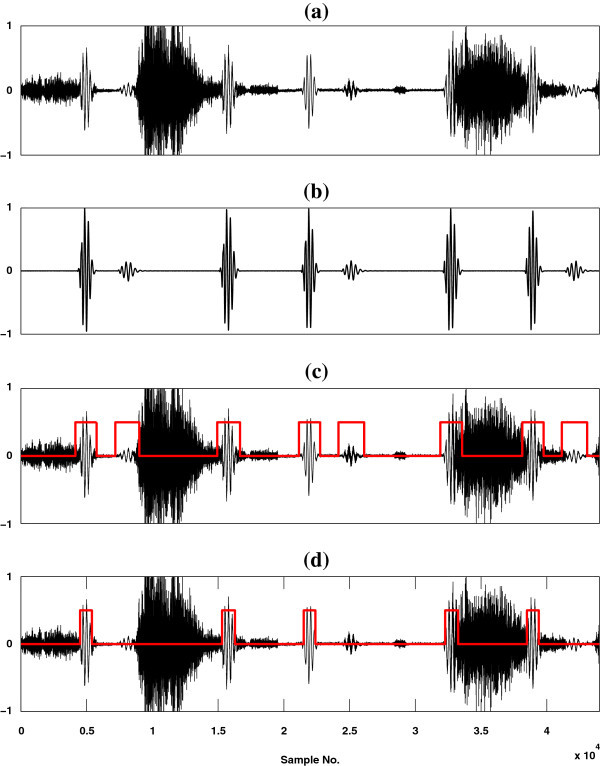
**Localization results of S1, S2 and S3 HS components.** **(a)** A high flow simulated mixed signal of heart and lung sounds. **(b)** Heart sound consists of S1, S2 and S3 components. **(c)** Output of HHA method (black curve corresponding to mixed sound data and red curve for heart sound region) and **(d)** depict the output of SSA method (black curve corresponding to mixed sound and red curve for heart sound segment).

**Figure 4:**
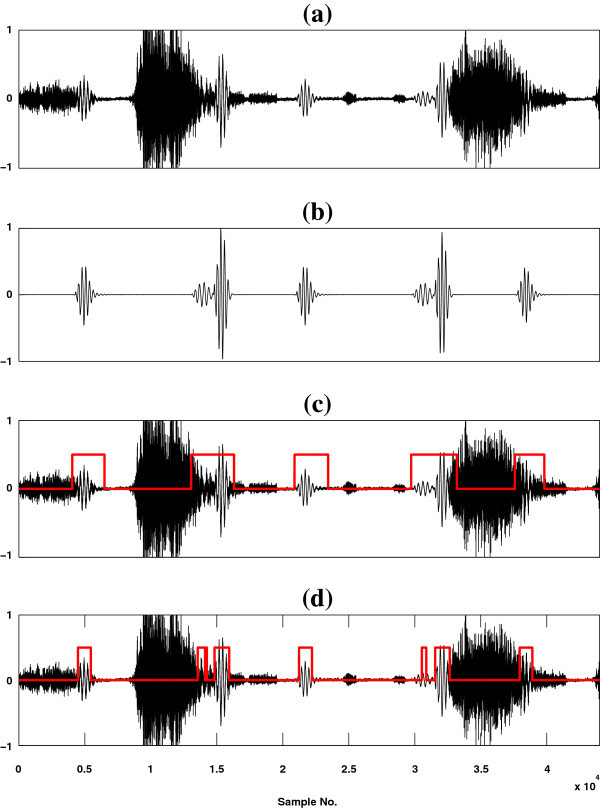
**Localization results of S1, S2 and S4 HS components.** **(a)** A high flow simulated mixed signal of heart and lung sounds. **(b)** Heart sound consists of S1, S2 and S4 components. **(c)** Output of HHA method (black curve corresponding to mixed sound data and red curve for heart sound region) and **(d)** depict the output of SSA method (black curve corresponding to mixed sound and red curve for heart sound segment).

**Figure 5:**
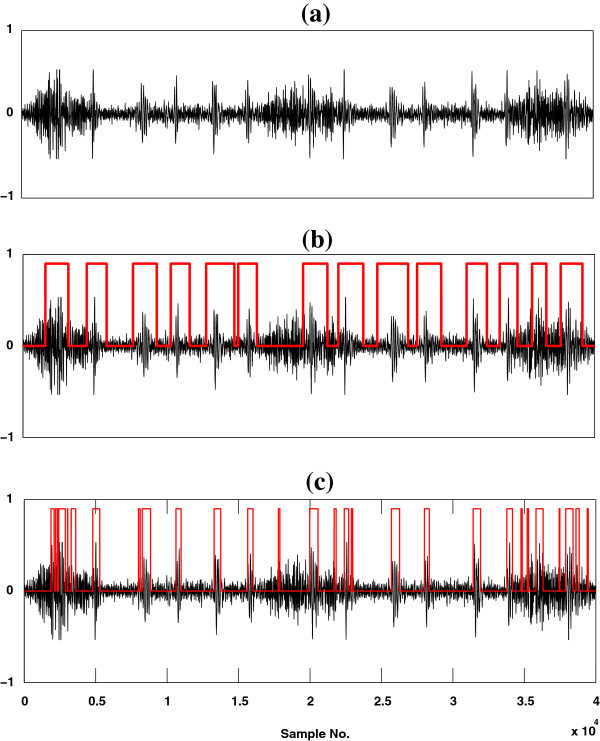
**Results of different heart sound localization methods for real recorded lung sound data.** **(a)** High flow real LS signal, **(b)** shows the output of HHA method and **(c)** displays the result of the SSA method.

The effectiveness of the proposed method is evaluated qualitatively through a visual display of the results and compared with the baseline method (SSA). Figure 3 shows a graphical representation of the results for a simulated high flow mixed LS signal [Figure 3(a)] along with a reference HS signal which consists of S1, S2 and S3 components [Figure 3(b)] and the outputs of these two methods [Figures 3(c-d)]. On the other hand, Figure 4 shows a graphical representation of the results for a simulated high flow mixed LS signal [Figure 4(a)] along with a reference HS signal which consists of S1, S2 and S4 components as shown in Figure 4(b) and the outputs of these two methods are depicted in Figures 4(c-d). In spite of these two figures, a pictorial illustration of the results for a real time recorded high flow mixed LS signal is shown in Figure 5 (a) along with the outputs of the two referred methods [Figures 5(b-c)]. From Figures 3(c-d) and Figures 4(c-d), it is seen that the proposed method detects the HS segments more correctly than the SSA method. The baseline method is incompetent to detect the third (S3) heart sound component as shown in Figure 3(d). In contrast to the baseline method, the proposed method (HHA) is competent to detect the S1, S2, S3 and S4 components of heart sounds as shown in Figure 3(c) and Figure 4(c). From the Figures 3(d), 4(d) and 5(c), it is observed that some part of HS included segment is not estimated and some non HS segment is detected as HS by SSA technique. On the basis of visual inspection, it is seen that the proposed method estimates HS boundary which is relatively larger in size than the reference HS boundary, but SSA method estimates HS boundary which is relatively less in size than the reference HS boundary. The performance of the proposed method is measured by comparing its output HS boundary with the reference HS boundary. In this study, the boundary of reference HS signal is calculated by three expert physicians based on auditory test and visual inspection of spectrogram and waveform of the reference HS signal.

### Quantitative evaluation of the results

A quantitative comparisons of these two methods are given in Tables 2, 3, 4, 5, 6. Tables 2, 3, 4, 5, present the results for normal and pathological simulated data and Table 6 gives results for real recorded data. The FNR, ACC, and DER of the proposed method are significantly better than the SSA method for various types of mixture (Tables 2, 3, 4, 5,) and real LS data (Table 6) at different flow rates. Moreover, the proposed method is faster than the SSA method. On the other hand, SSA method is better in term of FPR. However, the performance of any heart sound reduction technique which follows a preprocessing step of HS localization, depends on a correct estimation of HS segments. So, it is more important to estimate the segment that contains HS information than the detection of non HS segment as HS segment. The FNR value of SSA method and FPR value of the proposed method are greater for pathological HS signal than the normal HS signal due to the presence of murmur in cardiac sound signal. The performance of the two methods degrades gradually with increase of flow rate because of the superimposition of LS over HS. The SSA method gives a higher valued FNR for medium and high flow pathological signals. To overcome these difficulties, two modifications have been done with SSA technique: (1) first two principal components are chosen to reconstruct the HS dominated time series because the eigenvalue spectra have a bending point at the 2*nd* pair and (2) the threshold value and cutoff frequency of high pass filter mentioned in (Ghaderi et al. 2011) are reduced.

**Table 2:** **Performance of the different HS localization methods, HHA and SSA for the synthetic mixtures of normal HS and normal LS at three different flow rates**

TF	Method	Error (%)		ACC	DER	ET
		FNR	FPR	(%)	(%)	(Sec)
L	HHA	0.0 ± 0.0	1.05 ± 0.04	99.15 ± 0.03	0.83 ± 0.03	0.38 ± 0.01
L	SSA	24.63 ± 0.18	0.0 ± 0.0	96.05 ± 0.02	3.94 ± 0.02	1.39 ± 0.01
M	HHA	0.00 ± 0.0	2.42 ± 0.34	98.00 ± 0.27	1.99 ± 0.27	0.38 ± 0.01
M	SSA	28.10 ± 0.18	0.0 ± 0.0	95.49 ± 0.03	4.49 ± 0.03	1.39 ± 0.01
H	HHA	0.0 ± 0.0	5.46 ± 0.31	95.63 ± 0.23	4.35 ± 0.23	0.38 ± 0.01
H	SSA	34.90 ± 0.14	0.0 ± 0.0	94.40 ± 0.05	5.59 ± 0.05	1.39 ± 0.01

TF: Types of Flows; FNR: False Negative Rate; FPR: False Positive Rate; ACC: Accuracy; DER: Detection Error Rate; ET: Execution Time.

**Table 3:** **Performance of the different HS localization methods, HHA and SSA for the synthetic mixtures of normal HS and abnormal LS at three different flow rates**

TF	Method	Error (%)		ACC	DER	ET
		FNR	FPR	(%)	(%)	(Sec)
L	HHA	0.0 ± 0.0	1.85 ± 0.30	98.46 ± 0.25	1.53 ± 0.25	0.38 ± 0.01
L	SSA	25.80 ± 0.49	0.0 ± 0.0	95.86 ± 0.08	4.13 ± 0.08	1.39 ± 0.01
M	HHA	0.00 ± 0.0	2.86 ± 0.31	97.49 ± 0.14	2.49 ± 0.14	0.38 ± 0.01
M	SSA	31.85 ± 0.07	0.0 ± 0.0	94.88 ± 0.15	5.10 ± 0.15	1.39 ± 0.01
H	HHA	0.0 ± 0.0	7.11 ± 0.08	94.41 ± 0.26	5.57 ± 0.26	0.38 ± 0.01
H	SSA	37.09 ± 1.94	2.27 ± 1.66	92.11 ± 2.74	7.88 ± 2.74	1.39 ± 0.01

TF: Types of Flows; FNR: False Negative Rate; FPR: False Positive Rate; ACC: Accuracy; DER: Detection Error Rate; ET: Execution Time.

**Table 4:** **Performance of the different HS localization methods, HHA and SSA for the synthetic mixtures of abnormal HS and normal LS at three different flow rates**

TF	Method	Error (%)		ACC	DER	ET
		FNR	FPR	(%)	(%)	(Sec)
L	HHA	0.0 ± 0.0	3.05 ± 0.32	97.92 ± 0.21	2.06 ± 0.21	0.38 ± 0.01
L	SSA	25.92 ± 0.59	0.0 ± 0.0	91.06 ± 0.20	8.92 ± 0.20	1.39 ± 0.01
M	HHA	0.00 ± 0.0	4.94 ± 0.47	96.73 ± 0.30	3.25 ± 0.30	0.38 ± 0.01
M	SSA	32.98 ± 1.76	0.0 ± 0.0	88.63 ± 0.60	11.36 ± 0.60	1.39 ± 0.01
H	HHA	0.0 ± 0.0	14.07 ± 1.25	91.57 ± 0.65	8.41 ± 0.65	0.38 ± 0.01
H	SSA	44.27 ± 3.30	2.64 ± 1.84	81.38 ± 0.92	18.59 ± 0.92	1.39 ± 0.01

TF: Types of Flows; FNR: False Negative Rate; FPR: False Positive Rate; ACC: Accuracy; DER: Detection Error Rate; ET: Execution Time.

**Table 5:** **Performance of the different HS localization methods, HHA and SSA for the synthetic mixtures of abnormal HS and abnormal LS at three different flow rates**

TF	Method	Error (%)		ACC	DER	ET
		FNR	FPR	(%)	(%)	(Sec)
L	HHA	0.0 ± 0.0	4.43 ± 0.60	97.01 ± 0.30	2.97 ± 0.30	0.38 ± 0.01
L	SSA	27.15 ± 1.26	0.0 ± 0.0	90.42 ± 0.47	9.56 ± 0.47	1.39 ± 0.01
M	HHA	0.00 ± 0.0	6.81 ± 0.39	95.59 ± 0.23	4.39 ± 0.23	0.38 ± 0.01
M	SSA	34.83 ± 0.95	0.83 ± 0.20	87.44 ± 0.25	12.54 ± 0.25	1.39 ± 0.01
H	HHA	0.0 ± 0.0	16.37 ± 0.72	90.40 ± 0.35	9.58 ± 0.35	0.38 ± 0.01
H	SSA	45.34 ± 0.64	3.97 ± 0.76	79.25 ± 1.69	20.73 ± 1.69	1.39 ± 0.01

TF: Types of Flows; FNR: False Negative Rate; FPR: False Positive Rate; ACC: Accuracy; DER: Detection Error Rate; ET: Execution Time.

**Table 6:** **Performance of the different HS localization methods, HHA and SSA for the real time recorded lung sounds at three different flow rates**

TF	Method	Error (%)		ACC	DER	ET
		FNR	FPR	(%)	(%)	(Sec)
L	HHA	0.0 ± 0.0	2.50 ± 0.33	98.15 ± 0.21	1.83 ± 0.21	0.23 ± 0.01
L	SSA	25.84 ± 0.99	0.69 ± 0.47	95.42 ± 0.78	4.56 ± 0.78	1.45 ± 0.01
M	HHA	0.0 ± 0.0	5.83 ± 0.10	95.59 ± 0.33	4.40 ± 0.33	0.23 ± 0.01
M	SSA	29.19 ± 0.52	1.24 ± 0.97	92.96 ± 0.87	7.03 ± 0.89	1.45 ± 0.01
H	HHA	0.0 ± 0.0	11.66 ± 0.80	93.05 ± 0.59	6.94 ± 0.59	0.23 ± 0.01
H	SSA	36.25 ± 0.61	1.53 ± 1.44	90.40 ± 1.68	9.59 ± 1.68	1.45 ± 0.01

TF: Types of Flows; FNR: False Negative Rate; FPR: False Positive Rate; ACC: Accuracy; DER: Detection Error Rate; ET: Execution Time.

### Effect of cut off frequency (*f*_*c*_) on the performance of the proposed method

**Figure 6:**
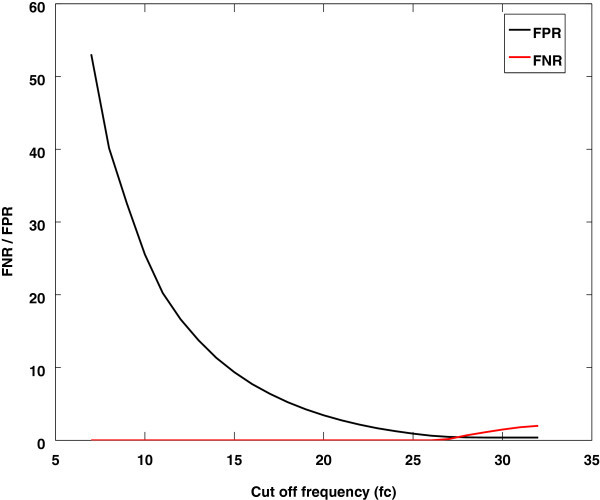
**The variation of FNR and FPR values with cut off frequency** ***f***_***c***_ **for a typical normal artificial mixed lung sound.**

**Figure 7:**
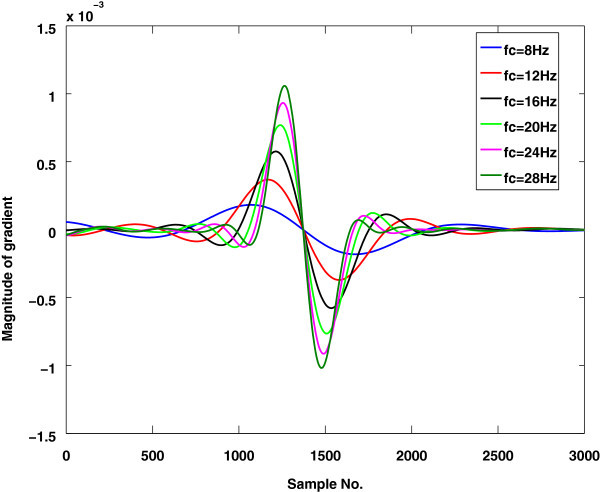
**The effect of cut off frequency** ***f***_***c***_ **on the calculation of extrema points.**

In this experiment cutoff frequency *f*_*c*_ of LPF used for the smoothing of the envelope signal, is set in the range of 7–25 Hz. In fact the performance of the method is directly influenced by *f*_*c*_. The FNR increases and FPR decrease with increasing *f*_*c*_ as shown in Figure 6. The reason for increment of FNR and decrement of FPR for high *f*_*c*_ is the shifting of minima points toward the maximum points. This occurs because of the presence of high frequency components in the filtered envelope signal as shown in Figure 7. In other words the estimated HS boundary is smaller than the actual HS boundary. The actual HS boundary is validated through several tests: auditory, visual inspection, spectrogram analysis, and WaveSurfer toolkit. The efficiency of the proposed method may be improved to a higher degree by deriving an optimum *f*_*c*_ value based on an adaptive filter. This issue may be addressed in a future work. The analysis of the results shows that the performance of HS localization algorithms is affected by flow rates and by pathological states. In spite of these shortcomings, the proposed method is superior than other technique in all aspects except FPR.

## Conclusion

A new HS localization algorithm is proposed in this work. This method is developed by using the Hilbert transform for envelope detection and Heron’s formula for area calculation. Here, HS segments are estimated by comparing their area with an adaptive threshold value. The performance of the method is compared with the SSA method described in (Ghaderi et al. 2011). The results are obtained by implementing the proposed and SSA method on simulated and real recorded LS data. In this study, different flow rate and various pathological conditions are considered. The results for simulated and real data show that the proposed method superior in terms of FNR, ACC, DER, and ET. However, the SSA method is better in term of FPR. The proposed technique gives a false negative rate of zero for all cases under all conditions and is faster. Hence, it is expected to have a high impact in real-life applications that interpret lung sounds.
